# “Showing the data” in published biology research

**DOI:** 10.1128/mbio.00572-26

**Published:** 2026-05-08

**Authors:** Benjamin G. Freeman, Marina E. Haldopoulos, Sidharth Srinivasan, Taylor L. Cooper, Soobin An, Miharu Yasumuro, Aiswarya Venkata Suresh Kumar, Melody Modarressi, Xinyu Zhang, Akul Chopra

**Affiliations:** 1 School of Biological Sciences, Georgia Institute of Technology 1372, Atlanta, Georgia, USA; 2 Interdisciplinary Graduate Program in Quantitative Biosciences, Georgia Institute of Technology 1372, Atlanta, Georgia, USA; 3 Wallace H. Coulter Department of Biomedical Engineering, Institute for Bioengineering and Bioscience, Georgia Institute of Technology 1372, Atlanta, Georgia, USA; Georgia Institute of Technology, Atlanta, Georgia, USA

**Keywords:** figure, graphic, data visualization

## Abstract

Showing the data is the first rule of effective figures, yet this mandate is often ignored. Perhaps the signature offender is the “dynamite plot”—a bar graph showing mean and error. Here, we evaluate recent trends in the use of dynamite plots by analyzing 8,834 figures from 2,930 studies published between 2021 and 2025 in 18 journals from five fields of biology. We find that dynamite plots constitute ~25% of figures and are especially common in microbiology journals. However, the use of dynamite plots has declined substantially across fields and journals from 30% of figures in 2021 to 18% in 2025—evidence that biologists increasingly show the data in their figures. We advocate for authors, reviewers, and editors to continue this trend, suggest simple dot plots as an effective replacement for dynamite plots, and describe other options when space or sample size makes dot plots less feasible.

## PERSPECTIVE

*“Above all else, show the data”*—Edward Tufte (1)

Scientists create figures to communicate patterns in their data to readers, but not all figures are created equal. As Tufte wrote, the first and foremost job of figures is to show the underlying data so that readers can evaluate patterns in the data. Yet, the widely used bar graph noting mean and error—the “dynamite plot”—directly violates this principle.

**Fig 1:**
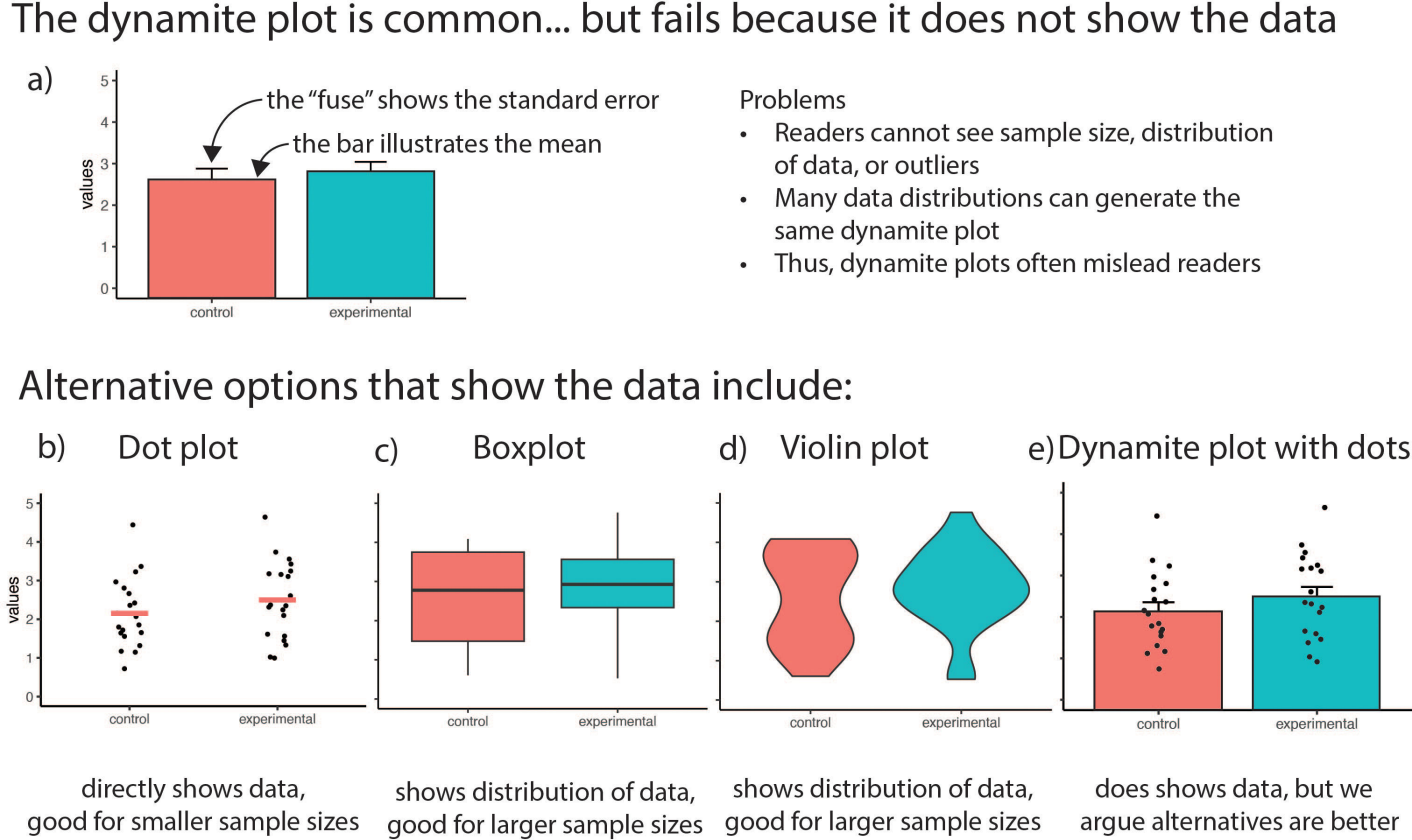
Dynamite plots (**a**) vs alternative plots (**b–e**) that illustrate the relationship between one categorical variable and one continuous variable. All example plots were sampled from the same data distribution. Dot plots (**b**) show the data points; the mean of each group can also be illustrated, as shown here with a red line. Boxplots and violin plots (**c and d**) show the distribution of the data when sample sizes are large and thus fulfill the mandate to show the data; if desired, individual data points can also be plotted atop boxplots or violin plots. Dynamite plots with dots overlain (**e**) do show the data, but the inclusion of the bar adds a little value compared with a simple dot plot.

Dynamite plots are a type of bar plot that shows the relationship between a continuous variable and a categorical variable (Fig. 1a). These plots illustrate the mean value of the continuous variable as the height of the bar and a measure of variation (often the standard error of the mean, sometimes the standard deviation) as a line extending above the bar, akin to a stick of dynamite with a fuse. A long list of scientists has pointed out that dynamite plots should be avoided because they do not show the data and conceal both sample sizes and the distribution of data (2–7). Failure to show the data leads to incorrect interpretations. Readers glancing at a dynamite plot often interpret sample sizes to be large and variables to be normally distributed; both assumptions are often wrong (4, 8, 9).

Despite these known disadvantages, dynamite plots remain commonly used across scientific fields (6, 10, 11). For example, dynamite plots are used in around half of the published articles in vascular disease journals (6) and surgical journals (11). Moreover, it remains unclear if the widespread calls to abandon the dynamite plot have affected change despite the existence of better alternative figure types (Fig. 1b through d and Fig. 1e): the incidence of dynamite plots remained roughly stable between 2010 and 2020 across a broad sample of scientific fields (10), though it declined somewhat within certain disciplines (11).

Here, we evaluate recent trends in the use of dynamite plots in biological research by analyzing 8,834 figures from 2,930 studies published between 2021 and 2025 in 18 journals representing five fields of biology. Our goal was to assess the degree to which biologists show the data in their figures and to determine whether the use of dataless dynamite plots has changed over the past 5 years.

**Fig 2:**
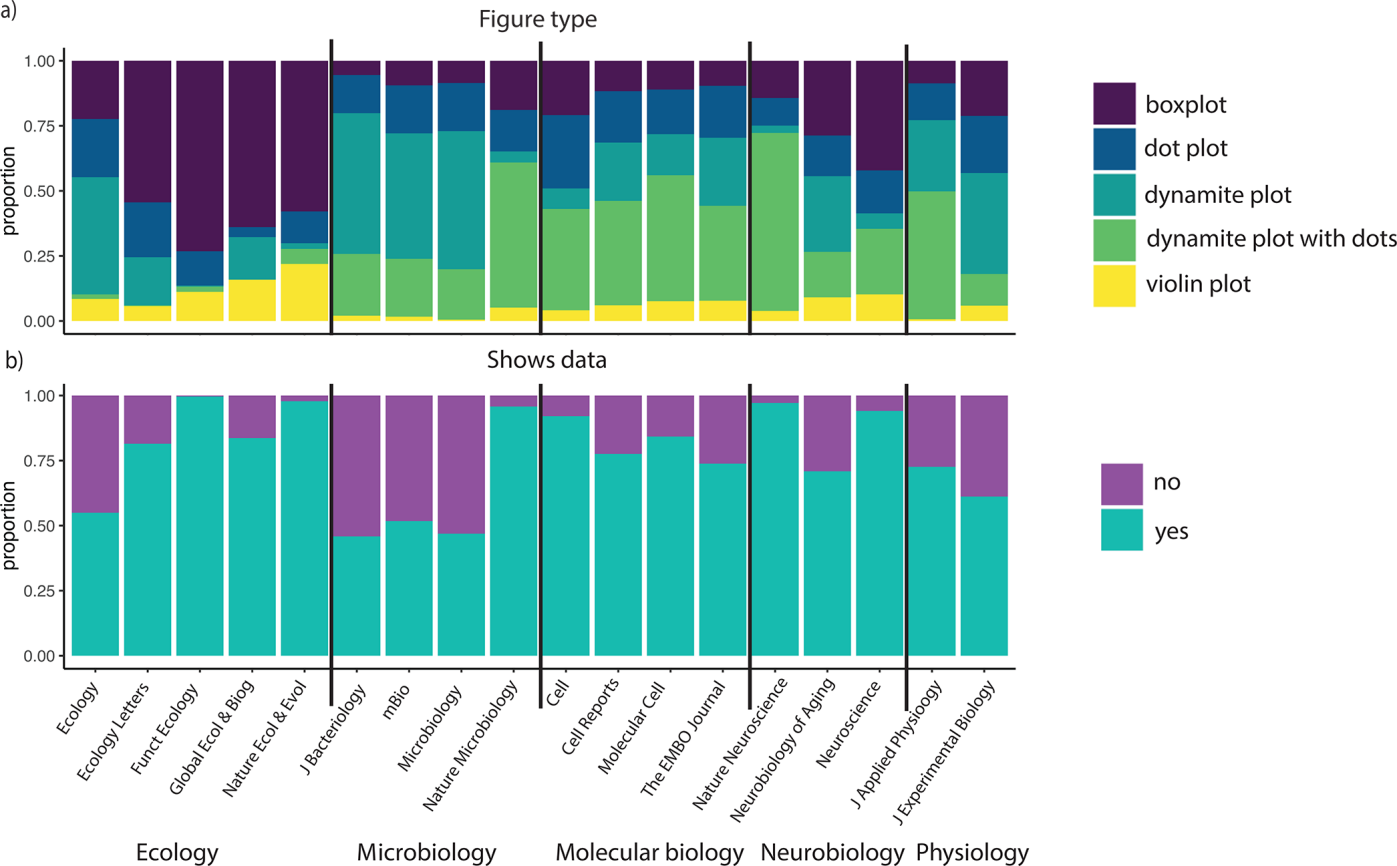
The proportions of figure types (**a**) and figures that show or do not show the data (**b**) published between 2021 and 2025 vary across 18 journals from five fields of biology.

Dynamite plots remain commonly used across biology. Overall, dynamite plots with dots were the most common graph type in our data set (32%), followed by dynamite plots (25%), boxplots (20%), dot plots (17%), and violin plots (6%). Individual journals vary in the proportion of figure types they contain, and some general differences between fields are apparent (Fig. 2a). For example, boxplots are common in ecology journals (53% of figures in these journals), dynamite plots are common in microbiology journals (37% of figures in these journals), and dynamite plots with dots are common in molecular biology journals (43% of figures in these journals). Overall, 75% of the figures we examined show the data. This proportion varied by field and journal (Fig. 2b), and it was higher for neurobiology (90%), molecular biology (82%), and ecology (82%) than for microbiology (63%) or physiology (68%; note that we only examined two physiology journals).

**Fig 3:**
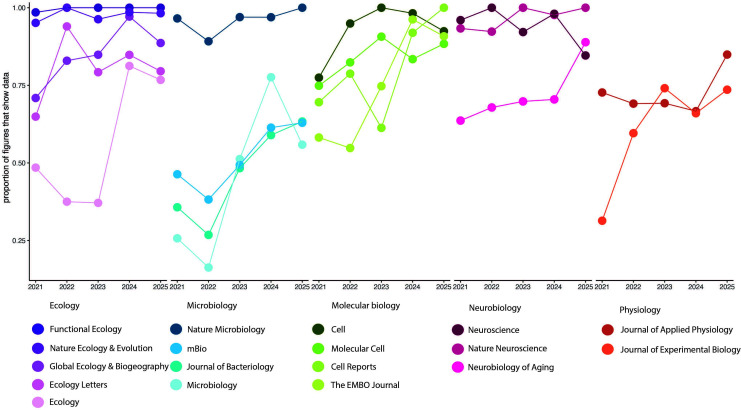
The proportion of figures that shows the data increased between 2021 and 2025 within sampled journals. These increases were widespread, occurring in 17 out of 18 journals, and were often substantial, especially for journals with initially low proportions of figures that show the data.

**Fig 4:**
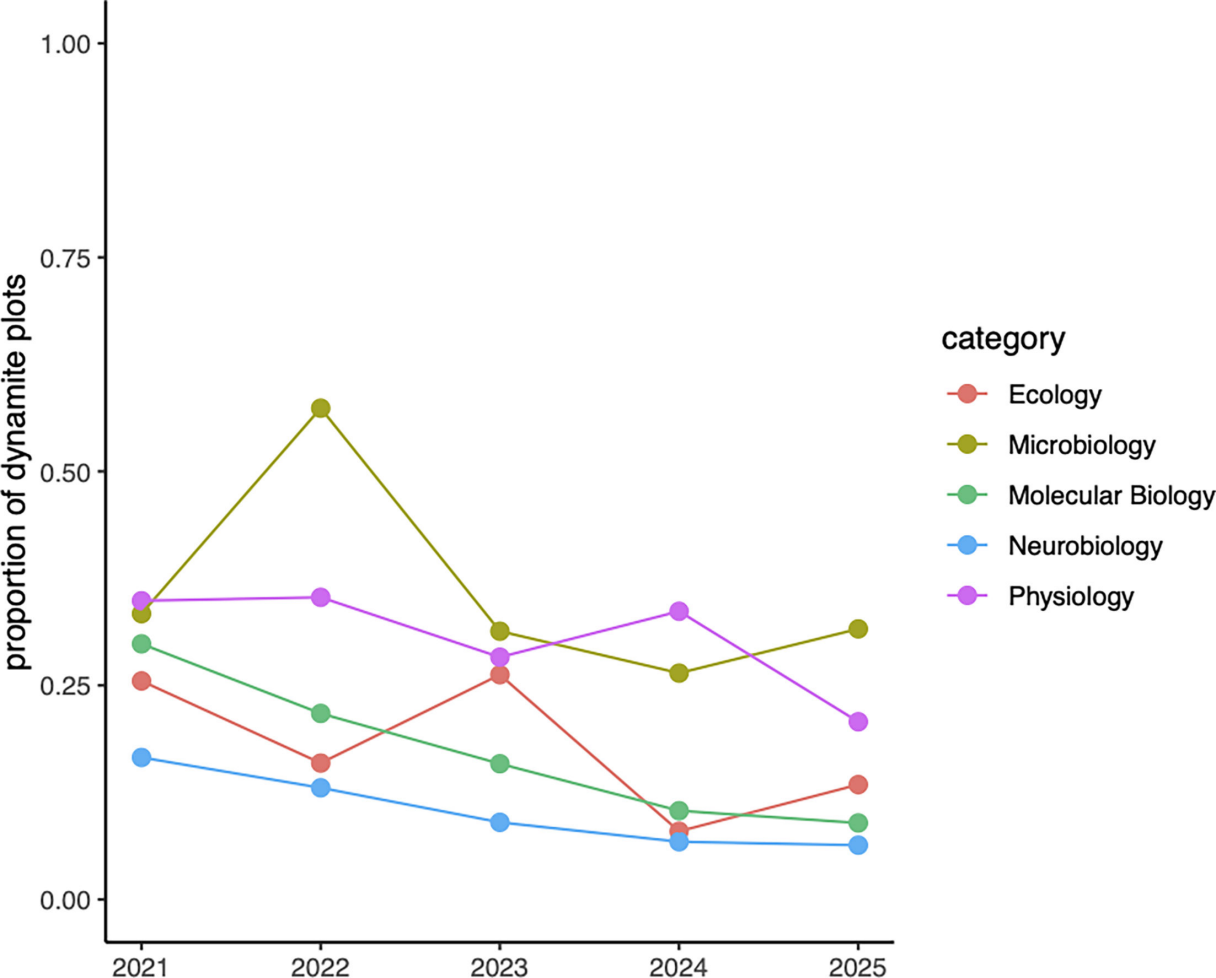
Biologists published fewer figures with dynamite plots between 2021 and 2025. This decline holds true across all five surveyed fields of biology.

We found a clear signal that biologists increasingly show the data in their figures within the sampled timeframe (Fig. 3). The overall proportion of figures showing the data increased from 70% in 2021 to 82% in 2025. Increases over this time frame were consistent across journals: 17 out of 18 journals had a higher proportion of figures that showed the data in 2025 compared to 2021, and many of these increases were substantial. Dynamite plot use declined from 30% of figures in 2021 to 18% in 2025 (Fig. 4); some of this decline is associated with an increase in the use of dynamite plots with dots, from 31% in 2021 to 36% in 2025.

Our survey of published figures across a sample of biology journals can be interpreted as a glass half-full or glass half-empty. An optimist might enthuse that biologists have greatly reduced their use of dynamite plots that do not show the data. At the same time, a pessimist would point out that dynamite plots remain a common graph type and appear to be especially popular in microbiology journals. We wish to highlight the optimistic perspective: biologists have made substantial progress in improving their graphical representation of data across fields in the short time span of the past 5 years.

At the same time, the pessimistic perspective deserves consideration. Among the journals we analyzed, conventional dynamite plots remain common, especially in microbiology journals, and dynamite plots with dots are common in many journals, particularly in molecular biology. How might biologists better show their data? One possibility is that top-down changes at journals can be an effective mechanism of change. Our data provide some support for this idea. The journals we surveyed that are part of the Nature Publishing Group had nearly 100% of their figures showing the data, perhaps because editors enforce the publisher’s stated requirement that authors show individual data points. It would be a worthwhile experiment for other editorial boards and publishers to enforce similar requirements.

A second pessimistic message is that while dynamite plots have declined over time, dynamite plots with dots are the most common figure type. These plots succeed in showing the data, but we argue that better options exist. For example, when sample sizes are small, a simple dot plot (perhaps with the mean illustrated, as in Fig. 1b) is typically more effective than a dynamite plot with dots. This is because the inclusion of the bar and measure of variation (the stick of dynamite and fuse) add visual elements that provide minimal additional information and may even mislead. Why not simply show the data points? When sample sizes are large, boxplots or violin plots are more effective at showing the distribution of the data than a dynamite plot with dots.

Our analysis provides useful data to evaluate recent trends in the use of different figure types within biology but also comes with limitations. For example, we decided to manually score figures to be as accurate as possible. Consequently, our sample size of figures was large but not overwhelming; the continued use of machine learning models that enable researchers to extract data from larger sets of figures is a promising approach (10, 11). We also sampled a modest number of biology journals from a handful of fields, with varying sample sizes of assessed figures among journals. Nonetheless, we argue that the journals we scanned provide a reasonable sample of biological journals that should allow us to detect overall trends within the broader biological publishing landscape.

In conclusion, we find that the dataless dynamite plot has become increasingly rare within biological journals in the past half decade. This decline is not preordained; scientists have long been admonished to show their data, and previous studies reported only modest declines in the use of dynamite plots between 2010 and 2020 across a large set of scientific fields (10). Thus, our findings suggest that biologists—editors, reviewers, mentors, and authors—increasingly fulfill Tufte’s mandate to show the data.

## METHODS

### Building a data set

We conducted this study as part of a graduate course on scientific writing during the fall semester of 2025 at the Georgia Institute of Technology; students in this course represented a variety of fields within biology. The instructor (B.G.F.) discussed principles of effective figures in class, admitted to publishing figures with dynamite plots in his scientific youth, and then invited students to participate in this project. Participating students were assigned to extract data from the figures published in 2021–2025 in multiple representative journals within their field of biology. We included the following disciplines: molecular biology, ecology, microbiology, neurobiology, and physiology. We aimed to scan at least four general journals within each field. In the end, we collected data from 18 journals in molecular biology (*Cell*, *Cell Reports*, *Molecular Cell*, and *The EMBO Journal*), ecology (*Ecology*, *Ecology Letters*, *Functional Ecology*, *Global Ecology and Biogeography*, and *Nature Ecology and Evolution*), microbiology (*Journal of Bacteriology*, *mBio*, *Microbiology*, and *Nature Microbiology*), neurobiology (*Nature Neuroscience*, *Neurobiology of Aging*, and *Neuroscience*), and physiology (*Journal of Applied Physiology* and *Journal of Experimental Physiology*).

Participants collected data for all figures that graphically showed the relationship between one categorical and one continuous variable. Participants began their scan in the first issue of the year and aimed to categorize a minimum of 50 figures from each year in each journal, often achieving substantially higher sample sizes. The final data set included an average of 490 figures per journal (range: 208–1,090). Total sample sizes for each field were microbiology: 3,313; molecular biology: 2,215; ecology: 1,518; neurobiology: 1,095; and physiology: 693. For each figure, participants recorded (i) the complete citation, (ii) the journal, (iii) the year, (iv) the figure number, (v) the figure type, (vi) whether data points were shown as points on the graph (yes/no), and (vii) notes. Following data collection, B.G.F. reviewed the data set and harmonized text descriptions of the figure into the following four categories: dynamite plots, dot plots, boxplots, and violin plots. Our overall goal was to evaluate patterns in how biologists show their data in figures. We consider that all dot plots, boxplots, and violin plots inherently show the data. We added a fifth category by subdividing dynamite plots into two categories: conventional dynamite plots that do not show the data and dynamite plots with data points overlaid as dots (dynamite plots with dots), which do show the data.

### Statistical analyses

We calculated the proportion of figures in each of the five categories overall, for each field of study, and for each journal. We then repeated this exercise by classifying figures as showing the data (dot plots, boxplots, violin plots, and dynamite plots with dots) vs not showing the data (conventional dynamite plots). Next, we examined trends over time in figures that show or do not show the data. To do this, we calculated the proportion of figures that show vs do not show the data per year at each of the three scales (overall, by field of study, and per journal). All analyses were conducted in R (R Development Core Team 2025).

## Data Availability

The data set and code to produce analyses and figures are available in Zenodo at https://zenodo.org/records/19891090

## EDITOR'S NOTE

**Ed. Note**: A potential conflict of interest was addressed during the review process, and a senior editor conducted an additional review to ensure the integrity and fairness of the peer review process.
